# Psychometric properties and cross-language equivalence of the revised Bristol Rheumatoid Arthritis Fatigue and the Rheumatoid Arthritis Impact of Disease scales in rheumatoid arthritis

**DOI:** 10.1007/s11136-019-02188-8

**Published:** 2019-04-26

**Authors:** Martijn A. H. Oude Voshaar, Christina Bode, Sarah Hewlett, John Kirwan, Laure Gossec, Mart A. F. J. van de Laar

**Affiliations:** 1 Department of Psychology, Health and Technology, University of Twente, PO BOX 50 000, 7500 KA Enschede, The Netherlands; 2 University of West England, Bristol, UK; 3 Sorbonne Université, Paris, France; 4 Rheumatology Department, Pitié Salpêtrière Hospital, APHP, Paris, France; 5 Department for Rheumatology, Arthritis Centre Twente, Medisch Spectrum Twente, Enschede, The Netherlands

**Keywords:** Item response theory, Differential item functioning, Patient reported outcome, Rheumatoid arthritis, Fatigue, Disease impact, Validity, Reliability

## Abstract

**Objective:**

To assess psychometric properties and cross-language measurement equivalence of six versions of the Bristol Rheumatoid Arthritis Fatigue Scale (BRAF-MDQ) and the Rheumatoid Arthritis Impact of Disease Score (RAID in rheumatoid arthritis (RA).

**Methods:**

Both questionnaires were completed by French (*n* = 206), German (*n* = 206), Dutch (*n* = 317), Spanish (*n* = 157), Swedish (*n* = 170) and UK (*n* = 210) RA patients. The presence of cross-language differential item functioning (DIF) was examined using the generalized partial credit model. The impact of DIF on the item and total scores was examined by comparing DIF unadjusted and DIF adjusted expected item and scale scores. IRT-based methods were used to assess psychometric properties of the instruments.

**Results:**

11 of the 20 BRAF-MDQ (55%) and 4 of the 7 RAID items (57%) exhibited significant DIF in at least one of the six countries. The mean number of items with DIF per country was 2.6 for BRAF-MDQ and 1.1 for RAID. However, the impact of DIF on the total RAID and BRAF-MDQ scores, as well as the BRAF subscales, was found to be negligible at the group level. Only for the BRAF physical subscale was there evidence of minor DIF. Marginal reliabilities of BRAF-MDQ (0.93) and RAID (0.89) were excellent, and precise scores could be obtained across the spectrum of disease impact and fatigue scores measured by these PROMs.

**Conclusion:**

This study supports the cross-language measurement equivalence of BRAF-MDQ and RAID and provides further support for the psychometric properties of these measures in RA.

**Electronic supplementary material:**

The online version of this article (10.1007/s11136-019-02188-8) contains supplementary material, which is available to authorized users.

## Introduction

Rheumatoid arthritis is an inflammatory joint disease, often with a chronic course that is known to impact patients’ quality of life in a variety of ways. Consequently, patient-reported outcomes such as pain and physical function have a prominent role in outcome assessment in this field [1, 2]. Other patient-reported outcomes, particularly fatigue and social role participation, have also gained increased attention [3, 4].

A variety of measures has been developed to facilitate the measurement of such PROs. For example, a patient-reported response index, the Rheumatoid Arthritis Impact of Disease (RAID) score, which combines 7 PRO domains, including fatigue, emotional well-being and sleep quality in one measure is now available and evidence regarding its measurement properties has been published [5, 6]. The multidimensional Bristol Rheumatoid Arthritis Fatigue scale (BRAF-MDQ) is a patient-reported outcome measure (PROM) that provides in-depth information about fatigue. Several studies have supported its measurement properties in RA [7–9].

Recently, these PROMs were cross-culturally adapted for use in 6 European countries, using a rigorous qualitative approach that focused on their linguistic and conceptual equivalence [10]. This work ensured that item content is appropriate for use in new cultural contexts and that the intended meaning of the involved items was retained in translation [11]. For BRAF-MDQ, a subsequent study showed that BRAF-MDQ yield reliable scores and that the same factor structure applied in each county [12]. These findings support the configural invariance of BRAF-MDQ scores across the countries considered, which suggests that in each of the considered countries the items measure the same constructs [13]. A next question to be addressed is to what extent these procedures have been successful in practice and hence the legitimacy of comparing BRAF-MDQ and RAID scores meaningfully across cultures. This requires that patients with the same overall fatigue or disease impact level can also be expected to have the same scores on the included items, regardless of the PROM language version administered to them [14]. Items for which this is not the case display differential item functioning (DIF). If multiple items in a scale are found to show DIF, the scale might systematically over or underestimate between country differences in the measured trait and scores cannot be meaningfully compared across different language versions, unless DIF is taken into account in the PROM scoring procedure [13, 14].

Item response theory (IRT) provides a framework for evaluating DIF, as well as the general scaling properties of a PROM. In IRT models for ordered polytomous data, the expected item responses for patients with different levels of the measured trait are described by an item characteristic function (ICF), which constrains the expected item scores to be monotonically increasing over the latent variable that the PROM intends to assess. Therefore, if an individual item in a scale shows good fit to an IRT model, it supports that the item is useful for measuring the latent variable the PROM intends to assess. Examining cross-language measurement equivalence using IRT involves testing whether the expected item scores of different language versions of a PROM item can be described using the same ICF. This would suggest that the item functions the same way in each language and would support that item scores can be meaningfully compared between patients who were assessed using different language versions of the scale. In cases where some PROM items are being responded to differently by patients of different languages, it is usually possible to improve the fit of the model by allowing country-specific ICF’s for DIF-affected items [17]. As long as there are sufficient numbers of DIF free items, the different language versions will still be in the same IRT metric. Modeling DIF in this way is an effective way to adjust the scores for DIF and preserve comparability of scores [18]. The impact of cross-cultural DIF on the comparability of the raw scores across different language versions of the PROM can be evaluated by examining the distance between an item’s unadjusted and DIF adjusted ICF’s on the latent variable or equivalently the differences between the adjusted and unadjusted predicted scale scores [19].

Once the items of a PROM have been successfully calibrated using an IRT model, the precision of the scores can be summarized using a marginal reliability coefficient, but can also be examined in detail, across the different trait levels discriminated between by the PROM using conditional reliability coefficients, which provides for a more in depth evaluation of score precision compared with classical test theory-based methods that are more commonly used.

The primary aim of the present study was to examine cross-language measurement invariance of RAID and BRAF-MDQ, using data from approximately 200 patients in each of 6 European countries for which the questionnaires had been translated [20]. We examined the presence of DIF and its impact on the item and scale levels using several effect sizes statistics that have been proposed for these purposes. A secondary aim was to examine measurement precision of the instruments.

## Methods

### Patients

A cross-sectional study [12] was performed in which consecutive patients with confirmed RA attending rheumatology clinics in France, Germany, The Netherlands, Spain, Sweden and the UK were invited to complete the BRAF-MDQ and RAID.

#### Measures

BRAF-MDQ is a PROM developed to provide detailed information about fatigue experienced by patients with RA. It contains 20 questions which can be summed to provide an overall fatigue score, with higher scores indicating more severe fatigue. The 20 items can also be used to calculate scores for Physical fatigue (items [1–4]), Living with fatigue (items [5–11]), Emotional Fatigue (items [12–16]) and Cognitive Fatigue (items [17–20]), again with higher scores indicating worse fatigue. BREAF-MDQ items 4–20 have a four response options rating scale ranging from “Not at all,” “A little,” “Quite a bit,” to “Very much,”. BRAF-MDQ item 1 is a 10-point numerical rating scale of overall fatigue severity. Item 2 asks respondents to report the number of days during which fatigue was experienced over the last weak and item 3 asks patients to indicate the average amount of time (less than 1 h, more than 1 h but not the whole day, the whole day) fatigue was experienced on the days with fatigue.

RAID is a PRO instrument that assesses the impact of RA across 7 domains (pain, functional disability, fatigue, sleep, coping, emotional and physical well-being). Each domain is assessed using a 11-point numerical rating scale, with higher scores indicating more disease impact. Domain scores are weighed by their importance according to patients and then combined to an overall disease impact score.

The Stanford Health Assessment Disability index (HAQ-DI) was administered to evaluate physical function [21]. HAQ-DI is one of the core set measures in RA and frequently used to characterize the general status of patients. HAQ-DI asks patients to rate the amount of disability they experience in 20 everyday activities on a scale ranging from 0 (without any difficulty) to 3 (cannot do). Items score are combined to produce 8 category scores. A total HAQ-DI score is obtained by averaging the category scores.

### Statistical analysis

#### Item response modelling

We considered the Rasch-based Partial Credit model (PCM) as well as a more general model, the Generalized partial credit model (GPCM) for item calibration. Both models are IRT models for ordered polytomous data suitable for use with items that have different numbers of response options. In these models the item responses by patients are explained by item and person parameters that are related to the latent variable that the PROM intends to assess. For both models, this latent variable can be imagined as a continuum on which each patient’s latent variable score is represented as a location, with higher values indicating higher fatigue or disease impact. Item characteristics are also mapped on the same continuum in the form of item threshold parameters these represent for each pair of adjacent response options, the latent variable score at which patients are equally likely to choose either response option. Furthermore, for each item threshold parameter, the probability that a patient chooses the higher of the adjacent response options is described using a logistic function of the distance between these parameters on the latent variable. In the PCM, all item response functions have the same slope, whereas for the GPCM an additional parameter is introduced which allows the slopes of items to differ.

In the first step of the IRT analysis, goodness of fit of the PCM and GPCM was compared using a likelihood ratio test for nested models. Marginal maximum likelihood estimates of the GPCM parameters and the means and variances of the different groups of respondents were obtained using the Multidimensional Item Response Theory (MIRT) package [22].

In the initial item response model, which will be referred to as the *unadjusted model*, a single set of item parameters is used to describe response behavior of patients in all countries. Like the BRAF-MDQ and RAID scoring rules, where item scores are combined to produce a single disease impact or fatigue score, this model does not account for differences in response behavior across countries.

Next, an item response model was created where item characteristics could be different in countries where evidence was found of cross-cultural differential item functioning (DIF). This model will be referred to as the *adjusted model* from here on. Lagrange Multiplier (LM) statistics and associated effect size statistics (described below) were used to identify items that exhibited DIF [17]. A scale purification procedure that involves assigning group-specific parameters to items flagged for DIF was used to model between country differences in response behavior [23–26]. This is an iterative procedure in which the item with the largest cross-cultural DIF according to the LM test is assigned group-specific items parameters first, and the model is rerun to see if bias in other items persists [18]. Iterations were stopped once the fit of the model could no longer be improved. Once completed, the scale purification procedure yields an item response model that can be used to obtain fatigue and disease impact scores in which cultural differences in response behavior are taken into account [27]. The fit of the adjusted model was evaluated using an LM test pertaining to the form of the item response curves [28].

#### Item- and scale-level impact of differential item functioning

A variety of ways to assess the impact of differential item and scale functioning are available, most of which rely on the concept of the expected item—(ES) and scale scores (ESS) [19]. ES are calculated as the sum of the probabilities of a response to each of an item’s *m* response options, times the scoring weight of that response option for any level of fatigue/disease impact. All these effect size statistics are sensitive to uniform and non-uniform DIF. Expected scale score (ESS) can be calculated as the sum of the expected item scores over all the items included in a scale.

At the item level, impact of DIF was assessed using an effect size statistic proposed by Glas, defined as the difference between average observed scores in the subpopulation under consideration and average expected scores for that subpopulation under the IRT model [17]. These statistics were divided by the maximum attainable item score so that for example 0.05 indicates that the observed average score was 5% different from its expectation under the model. For the RAID items, effect size statistics were calculated on the weighted item scores, as proposed in the original paper [5]. In all cases, cutoff values of 0.05 were maintained in the present study.

The scale-level impact of DIF in the present sample was examined using the signed test difference effect size statistics (STDS) proposed by Meade [19] which is calculated as the average difference ESS_u_–ESS_a_ across the sample of patients within a country under consideration. This effect size is compensatory (i.e., DIF potentially cancels out across items) and therefore is well suited to assess the actual impact that differential item functioning had on the mean fatigue and disease impact scores in this sample. To explore the maximum extent to which any patient’s score was impacted by DIF in the present sample, the “D-MAX” statistic proposed by Meade was also calculated which represents the largest absolute difference between the unadjusted and adjusted model’s expected response observed in the sample. Finally, we visually inspected the differences between the scale characteristic curves for the unadjusted and adjusted models across the range of possible fatigue/disease impact total scores. This analysis was performed to see if score bias occurred at infrequently observed disease impact/fatigue levels in the present sample.

#### Examination of psychometric properties

The standard errors associated with the different score levels of both PROMs were obtained for the range of the latent variable from − 5 to + 5. These were transformed to conditional reliability coefficients to describe measurement precision for different levels of the measured outcomes [29]. To describe the overall reliability of the scales, Marginal reliability coefficients were subsequently obtained by integrating the conditional reliabilities over the standard normal distribution [27, 30].

## Results

Patient characteristics are presented in Table 1. The number of included patients per country ranged from 157 (Spain) to 317 (The Netherlands). Across countries, patients had moderately severe disability with mean HAQ-DI scores ranging from 0.78 to 1.26 and disease impact levels, and fatigue scores were also in the moderately severe range.

**Table 1:** Patient characteristics

	France	Germany	The Netherlands	Spain	Sweden	UK
N	206	216	317	157	170	210
% Female	85.4%	69.9%	61.2%	87.9%	76.5%	78.6%
% 60 + years	42.7%	51.2%	61.7%	27.1%	64.7%	44.4%
HAQ, M (SD)	0.78 (0.61)	0.84 (0.74)	1.00 (0.61)	1.05 (0.61)	1,05 (0.61)	1.26 (0.80)
BRAF-MD, M (SD)	27.46 (16.55)	22.02 (14.39)	22.05 (13.98)	26.11 (16.74)	27.13 (16)	34.19 (17.28)
BRAF physical, M (SD)	11.66 (5.68)	10.44 (5.52)	11.39 (5.55)	11.29 (6.2)	12.5 (5.65)	14 (5.59)
BRAF living, M (SD)	6.55 (5.66)	5.79 (5.11)	5.3 (4.69)	6.55 (5.41)	6.55 (5.16)	8.69 (5.95)
BRAF cognition, M (SD)	4.37 (3.92)	3.47 (3.24)	3.09 (3.17)	4.66 (4.07)	4.68 (3.85)	6.25 (4.36)
BRAF emotion, M (SD)	4.56 (3.65)	2.36 (2.62)	2.24 (2.51)	3.55 (3.05)	3.46 (3.17)	5.02 (3.39)
RAID, M (SD)	3.78 (2.16)	3.65 (2.29)	3.71 (2.04)	4.39 (2.41)	4.46 (2.17)	5.24 (2.45)

### Analysis of cross-cultural differential item functioning

**Fig. 1:**
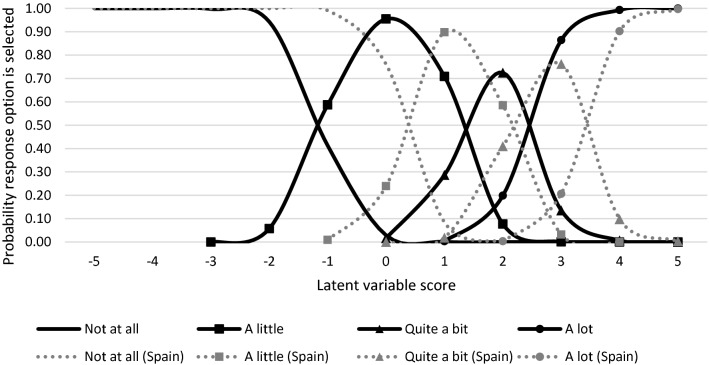
DIF in Spanish BRAF-MDQ item 18. Item response curves for item 18: Have you felt embarrassed because of your Fatigue. Each response curve reflects the probability that a respondent elects the pertaining response option across different levels of fatigue. 0 represents the average level of fatigue in the present sample.; solid lines refer to the item response model in the overall sample dotted lines refer to the item response model that applies to Spanish patients

First we compared the fit of the Rasch-based PCM and the two parameter GPCM. The results of the likelihood ratio test showed that the GPCM fitted significantly better for both the BRAF-MDQ *χ* (DF 50) = 1175, *p* < 0.01) and RAID models *χ* (DF 26) = 400, *p* < 0.01). Therefore, we proceeded with the GPCM in the remainder of the paper. The BRAF item: “Have you been embarrassed because of your fatigue?” was most strongly affected by DIF according to the LM test for DIF across countries (LM = 127.46, *p* < 0.01, ES = 0.05) Inspection of the ES per country for this item showed that response behavior was different in France (ES = 0.06), Spain (ES = 0.08), and Sweden (ES = 0.05), but not in the remaining countries (ES = 0.03 in all cases), see Table 2). Therefore, this item was split into virtual items for France, Spain, and Sweden and one item to represent the remaining countries. The nature of cross-cultural DIF of the Spanish version of this item is illustrated in Fig. 1 which presents a plot of the Spanish item response model for item 18, compared with the item response model estimated from the countries unaffected by DIF. It can be seen in the figure that the response curves shifted to the right of the fatigue IRT metric, which means that Spanish patients are in general less likely to report feelings of embarrassment related to their fatigue compared with patients from other countries. After DIF for BRAF item 18 was accounted for, the DIF analysis was repeated to see if DIF in other items persisted. In successive iterations of the procedure, DIF of respectively BRAF items 1 and 2 was addressed, followed by a number of items for which DIF was limited to a single country (Table 2). In the analysis of the RAID items, country-specific item parameters were first assigned to item 7, followed by items 3, 4, and 5. It should be noted that although a relatively large number of RAID items were assigned country-specific parameters, based on the significance of the LM test, the magnitude of DIF according to the ES statistics was quite minor for RAID items compared with BRAF items, with none of the items with ES > 0.05.

**Table 2:** Items assigned country-specific item parameters

	BRAF	RAID
France	18	5
Germany	1, 2, 9	3, 4
The Netherlands	5, 6, 7, 11	7
Spain	1, 2, 18	
Sweden	2, 12, 17, 18, 19	7
UK	1, 2	4, 7

In the next step of the analysis, fit of the adjusted model was evaluated. Results are presented in full in the supplemental material, together with the item parameters of the final models. There were three BRAF items and one RAID item with a statistically significant LM test in the GPCM calibration, which is slightly more than expected based on chance. However, only in case of the BRAF item 2: “How many days did you experience fatigue during the past week?” did the ES exceed the cutoff for substantial lack of fit of ES = 0.05. Therefore, our conclusion was that the DIF adjusted model fitted well. The item parameters of the adjusted models are presented in supplemental Table 1. Note that for some items the threshold parameters are not ordered in value in the same way as the response options. This is a well-known phenomenon that reflects that certain response options were unlikely to be selected by patients in this sample [31]. The ability of the items to discriminate between different levels of disease impact was quite varied, with discrimination parameters ranging from 0.47 to 2.1. The BRAF item about the number of days with fatigue (*α* = 0.47 in the unadjusted model) as well as RAID item 4 (sleep) was the only items that were found to be weakly related to the overall disease impact variable (*α* = 0.56).

### Scale-level impact of differential item functioning

**Fig. 2:**
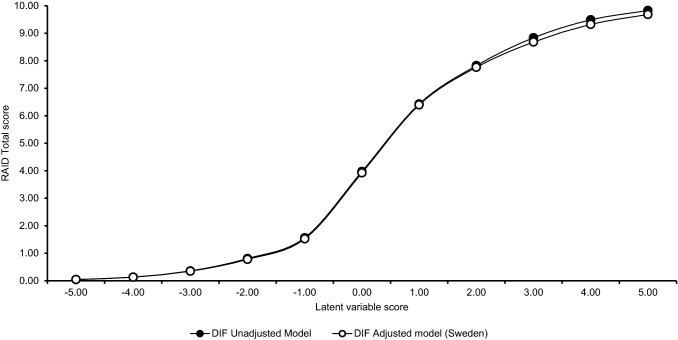
Predicted RAID total scores

Impact of DIF on the scale level was mostly minor with STDS ranging from 0 to 5% of the maximal attainable total score of the respective scale (Table 3). The means of the RAID and BRAF-total scores were largely unaffected with STDS < 1% of the maximum attainable total score. Slightly larger impact of bias was observed for some of the BRAF subscales with the means of the BRAF physical and emotional subscales affected most strongly. For example, according to the SIDS statistics, DIF adjusted scores were 1 scale point lower (i.e., STDS = 1.0) than unadjusted scores in the Spanish sub-sample. When looking at the most extreme observed difference between unadjusted and adjusted expected scores, it was again the case that for RAID and BRAF-total scores the impact of bias on individual scores was minor with D-MAX ranging from 1 to 4% of the maximum attainable scale scores. However, for BRAF-physical, the difference between adjusted and unadjusted scores was as high as 19% of the maximum attainable score for at least one German patient. D-MAX was also relatively high for Swedish patients responding to the BRAF-emotion scale, reflecting that 3 of the four items of this subscale were assigned country-specific item parameters. These results suggest that except for BRAF-physical and BRAF-emotion, bias did not have a strong impact on the cross-cultural comparability of the total scores for individual patients. Inspection of the scale characteristic curve did not reveal concerning differences between adjusted and unadjusted expected scores for infrequently occurring score levels for any instrument. This is illustrated for the RAID, in Fig. 2. Because each RAID NRS is weighed by an importance rating, ranging from 0.12 to 0.21, the RAID total scores range from 0 to 10. The figure shows that patients with an average disease impact level (Latent variable score = 0) are expected to have a RAID score of about 4, which correspond with the RAID scores in Table 1. Furthermore, according to the model, patients with disease impact score of − 2 (i.e., ~ 2 SDs below the mean) are expected to have a score of 0 and patients with a score of 3 are expected to score 10. These findings correspond well to the findings that 2.2% and 0.2% of patients had RAID scores of 0 and 10, respectively. Throughout the IRT measurement continuum, the predicted RAID scores were similar for the adjusted and unadjusted model.

**Table 3:** Impact of DIF on cross-cultural comparability of scores expressed using effect size statistics and (in parentheses) as percentages of the total score

	RAID* (0–10)			BRAF total (0–70)			BRAF physical (0–22)			BRAF living (0–22)			BRAF cognition (0–15)			BRAF emotion (0–12)		
	STDS	D-MAX	TCC	STDS	D-MAX	TCC	STDS	D-MAX	TCC	STDS	D-MAX	TCC	STDS	D-MAX	TCC	STDS	D-MAX	TCC
France	− 0.13 (1%)	0.43 (4%)	0.00 (0%)	0.41 (1%)	1.89 (3%)	0.34 (0%)	0.55 (3%)	2.03 (9%)	0.15 (1%)	− 0.02 (0%)	0.22 (1%)	0.01 (0%)	0.01 (0%)	0.17 (1%)	0.00 (0%)	0.25 (2%)	0.64 (5%)	0.12 (1%)
Germany	− 0.01 (0%)	− 0.30 (3%)	0.04 (0%)	− 0.20 (0%)	− 1.02 (1%)	0.34 (0%)	− 0.13 (1%)	− 4.09 (19%)	0.09 (1%)	− 0.13 (1%)	− 0.75 (4%)	0.16 (1%)	0.00 (0%)	0.17 (1%)	0.00 (0%)	− 0.07 (1%)	− 0.20 (2%)	0.01 (0%)
NL	− 0.03 (0%)	− 0.37 (4%)	0.28 (0%)	0.25 (0%)	1.56 (2%)	0.55 (1%)	0.48 (2%)	1.65 (8%)	0.15 (1%)	0.16 (1%)	0.67 (3%)	0.13 (1%)	0.01 (0%)	0.18 (1%)	0.00 (0%)	− 0.08 (1%)	− 0.20 (2%)	0.01 (0%)
Spain	0.05 (1%)	0.28 (3%)	0.06 (0%)	− 0.17 (0%)	− 1.43 (2%)	0.49 (1%)	− 1.0 (5%)	− 3.56 (16%)	0.24 (1%)	− 0.00 (0%)	− 0.22 (1%)	0.01 (0%)	0.01 (0%)	0.18 (1%)	0.00 (0%)	− 0.11 (1%)	− 0.42 (4%)	0.06 (1%)
Sweden	− 0.03 (0%)	− 0.43 (4%)	0.22 (0%)	0.38 (1%)	1.61 (2%)	0.75 (1%)	0.60 (3%)	2.03 (9%)	0.27 (1%)	− 0.00 (0%)	− 0.22 (1%)	0.01 (0%)	0.22 (1%)	0.43 (3%)	0.04 (0%)	− 0.01 (0%)	0.79 (7%)	0.18 (2%)
UK	− 0.04 (0%)	0.34 (3%)	0.16 (0%)	− 0.07 (0%)	− 1.72 (2%)	0.21 (0%)	− 0.29 (1%)	− 3.56 (16%)	0.14 (1%)	− 0.00 (0%)	− 0.23 (1%)	0.01 (0%)	0.00 (0%)	0.18 (1%)	0.00 (0%)	− 0.07 (1%)	− 0.20 (2%)	0.01 (0%)

BRAF: Bristol Rheumatoid Arthritis Fatigue scale; RAID: rapid assessment of impact of disease; results presented here pertain to the unweighted RAID total scores

#### Measurement properties

**Fig. 3:**
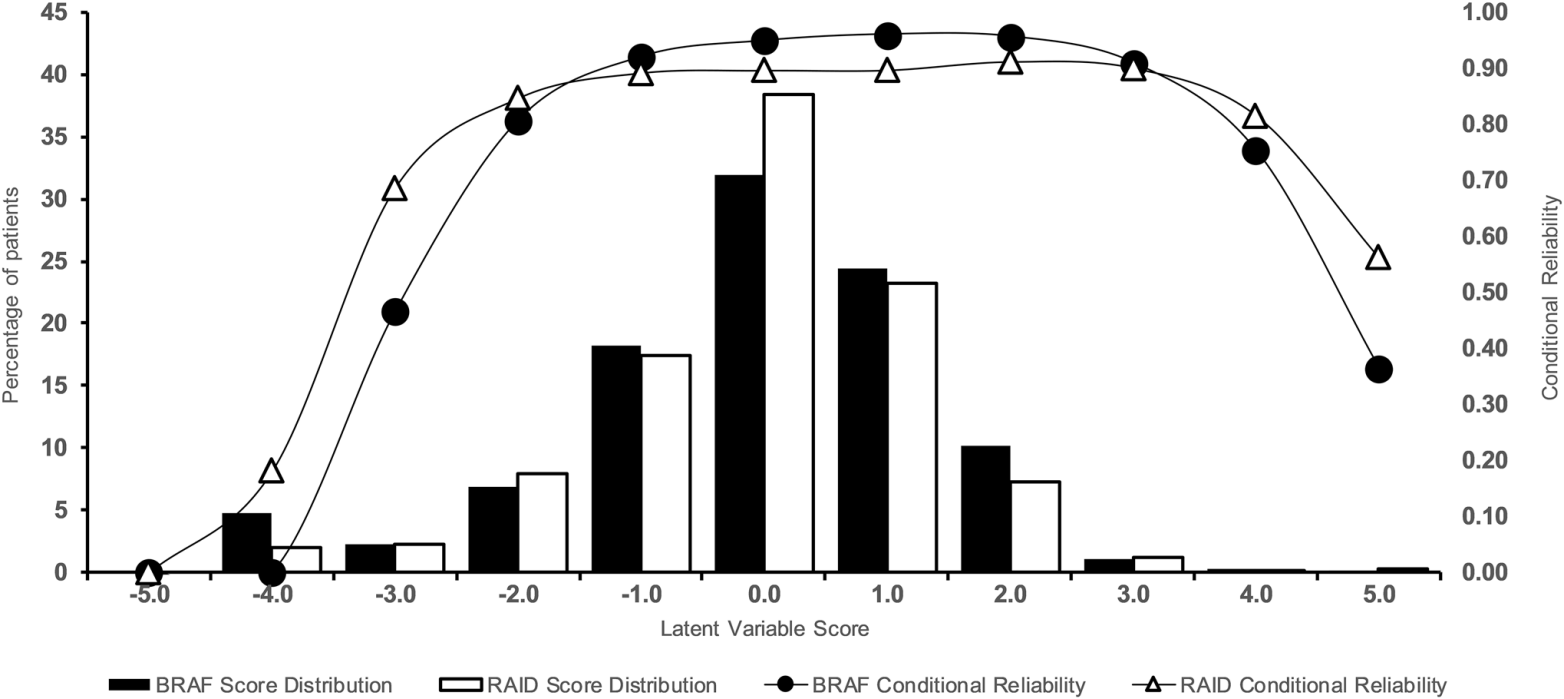
Local measurement precision

Both PROMS had excellent reliability, with marginal reliability coefficients of 0.89 for RAID and 0.93 for BRAF-MDQ. Figure 3 presents the conditional reliability coefficients, plotted over the distributions of respectively RAID and BRAF-MD Latent variable scores. Scores on both PROMs were approximately normally distributed with an Latent variable score (SD) of 0 (1). The figure shows that the items of both PROMs are well targeted to the RA patients in the present study; Reliability is highest for frequently occurring levels of fatigue/disease impact and the majority of patients in the present samples had a reliable score (i.e., conditional reliability > 0.70) for both the BRAF-MDQ (92%) and RAID (95%).

## Discussion

In the present study, we used IRT-based methods to evaluate the cross-language measurement equivalence and the psychometric properties of 6 European language versions of BRAF-MDQ and RAID. We found that although both instruments had a few items that exhibited language related DIF, accounting for these differences generally led to small differences in fatigue or disease impact estimates at the total score level. The results of this study therefore support the validity of BRAF-MDQ and RAID score comparisons between the different language versions considered in this study.

The BRAF-MDQ items 1 (NRS severity), 2 (How many days did you experience fatigue during the past week?) and 18 (Have you been embarrassed because of your fatigue?) and RAID item 7 (Coping) proved to be most consistently associated with DIF across countries. However, in subsequent analysis where these items were allowed to have country-specific characteristics, we observed good fit of the adjusted GPCM model. This finding supports the construct validity of the respective instruments; the same underlying variable of fatigue severity and disease impact, respectively, seems to apply to all items, but patients from different countries with the same level of fatigue/disease impact may have different expected item scores for some of the items.

The impact of language related DIF on the BRAF and RAID total scores was generally small, which suggests that raw scores can be compared between different language versions at the group level in most cases. However, for BRAF Physical in Spanish, Swedish, and French patients, the scores were inflated by ≥ 3% of the maximum attainable score. With respect to the scores of individual patients, the impact of cross-language factors was again generally quite minor. Only for BRAF physical, the impact of DIF was in some cases substantial so that the interpretation of BRAF physical scores of individual patients in a cross-cultural context should proceed with caution. Taken together, our findings provide support for the cross-cultural validity of RAID and BRAF-MDQ total scores.

However, in situations were small differences between different language versions of BRAF physical are sought, or when considering BRAF-physical scores of an individual patient assessed using different language versions, an IRT-based scoring procedure using the item parameters of the adjusted model might be prudent. Several IRT software packages can be used to estimate fatigue or disease impact scores based on the item parameters of the adjusted model, provided in Supplemental Material. The advantage of the IRT-based scoring procedures is that differences in item characteristics between different language versions are statistically adjusted, so that scores become better comparable across countries. The IRT-based scoring procedures are also more appropriate to use in case of missing individual item responses. However, the total sample size in this study was somewhat limited.

The psychometric properties of RAID and BRAF-MDQ have been described in several previous studies, using methods based on classical test theory. In these studies, both PROMs were found to have highly precise scores [5–8]. Our results corroborate the findings and expanded on them by showing the items are well targeted to the score levels of RA patients and that scores were precise across the spectrum of score levels. Finally, it has previously been demonstrated that the factor structure of BRAF-MDQ was stable across countries [12]. The findings support the configural of BRAF-MDQ scores across countries, i.e., that all items measure the same concepts in all countries. Our results expanded on this by demonstrating, for RAID and BRAF_MDQ that full measurement invariance was supported for both PROMs. Hence, scores can be meaningfully compared across different language versions. We also showed that all items could be described using the GPCM, which supports that the items relate to a common underlying variable. However, the analysis of IRT fit, together with the finding that the discrimination parameters varied quite a bit show that the Rasch model is not appropriate for these data. This means that the item responses contain more information about the disease impact/fatigue levels of patients then provided by the summed scores.

A limitation of the study is that convenience sampling was used to obtain samples of patients from different countries. Consequently, patients from different countries differed to an extent with respect to their fatigue, disease impact and HAQ scores which could have led to biased parameter estimates if a shared latent variable score distribution would have been assumed to apply to patients of all countries. In an effort to avoid this, we used separate marginal distributions to characterize the scores of each group of patients [32]. Furthermore, conclusions with respect to comparability of scores based on these s should not be generalized to other language versions of these instruments. For example, it might be expected that larger differences in item response behavior would have been observed for languages other than those belonging to the Indo-European family of languages and of versions for patients with different cultural backgrounds.

In summary, the results of this study generally support the validity of cross-cultural score comparisons using the instruments evaluated here and provide additional support for their measurement properties. Based on these results, we recommend the BRAF-MDQ and RAID total score as well as the BRAF living, emotion and cognition subscale for cross-cultural comparisons. Those interested in using BRAF physical in a cross-cultural context, we recommend using an IRT-based scoring procedure using the item parameters provided in the supplemental material.

## Electronic supplementary material

Below is the link to the electronic supplementary material.

Supplementary material 1 (DOCX 48 kb)
