# Supplementary material for: Psychometric properties and cross-language equivalence of the revised Bristol Rheumatoid Arthritis Fatigue and the Rheumatoid Arthritis Impact of Disease scales in rheumatoid arthritis
Source: Qual Life Res. 2019 Apr 26;28(9):2543–52. doi: 10.1007/s11136-019-02188-8 (PMC6698264; doi:10.1007/s11136-019-02188-8)
Supplement: Supplementary file 1 — Supplementary material 1 (DOCX 48 kb) [file 11136_2019_2188_MOESM1_ESM.docx]

|  | LM | Range LM | p | Range p’s | ES | Min ES | Max ES |
| --- | --- | --- | --- | --- | --- | --- | --- |
| BRAF-1 | 38.86 | (14.83-113.79) | 0.04 | (0.00-0.14) | 0.00 | -0.03 | 0.02 |
| BRAF-2 | 15.06 | (3.56-23.92) | 0.02 | (0.00-0.09) | 0.02 | 0.00 | 0.04 |
| BRAF-3 | 7.19 | (1.88-23.98) | 0.16 | (0.00-0.39) | 0.02 | 0.01 | 0.05 |
| BRAF-4 | 20.98 | (5.84-51.27) | 0.04 | (0.00-0.12) | 0.02 | 0.01 | 0.03 |
| BRAF-5 | 11.43 | (2.29-28.9) | 0.22 | (0.00-0.51) | 0.02 | 0.01 | 0.04 |
| BRAF-6 | 12.33 | (0.59-25.89) | 0.18 | (0.00-0.90) | 0.02 | 0.00 | 0.04 |
| BRAF-7 | 11.80 | (1.15-34.99) | 0.23 | (0.00-0.76) | 0.02 | 0.00 | 0.04 |
| BRAF-8 | 5.62 | (0.39-12.85) | 0.36 | (0.00-0.94) | 0.01 | 0.00 | 0.02 |
| BRAF-9 | 13.64 | (2.52-46.58) | 0.17 | (0.00-0.47) | 0.02 | 0.00 | 0.05 |
| BRAF-10 | 15.66 | (7.04-30.72) | 0.02 | (0.00-0.07) | 0.02 | 0.01 | 0.03 |
| BRAF-11 | 12.88 | (1.44-35.99) | 0.19 | (0.00-0.70) | 0.01 | 0.00 | 0.03 |
| BRAF-12 | 7.69 | (1.44-18.35) | 0.29 | (0.00-0.70) | 0.02 | 0.01 | 0.05 |
| BRAF-13 | 3.74 | (0.29-10.29) | 0.47 | (0.02-0.96) | 0.01 | 0.00 | 0.03 |
| BRAF-14 | 6.82 | (2.21-14.46) | 0.19 | (0.00-0.53) | 0.01 | 0.00 | 0.02 |
| BRAF-15 | 7.47 | (3.29-18.85) | 0.18 | (0.00-0.35) | 0.01 | 0.00 | 0.02 |
| BRAF-16 | 9.15 | (1.48-19.72) | 0.17 | (0.00-0.69) | 0.01 | 0.00 | 0.03 |
| BRAF-17 | 8.26 | (1.43-13.87) | 0.17 | (0.00-0.70) | 0.03 | 0.01 | 0.04 |
| BRAF-18 | 23.56 | (7.88-39.1) | 0.01 | (0.00-0.05) | 0.05 | 0.03 | 0.08 |
| BRAF-19 | 27.68 | (2.47-81.85) | 0.17 | (0.00-0.48) | 0.02 | 0.01 | 0.05 |
| BRAF-20 | 11.71 | (5.31-27.12) | 0.05 | (0.00-0.15) | 0.02 | 0.00 | 0.03 |
| RAID-1 | 31.06 | (12.81-66.8) | 0.06 | (0.00-0.23) | 0.01 | 0.00 | 0.01 |
| RAID-2 | 21.20 | (7.38-47.75) | 0.26 | (0.00-0.69) | 0.01 | 0.00 | 0.01 |
| RAID-3 | 21.11 | (5.12-37.99) | 0.23 | (0.00-0.88) | 0.01 | 0.00 | 0.02 |
| RAID-4 | 11.50 | (2.19-21.35) | 0.46 | (0.02-0.99) | 0.01 | 0.00 | 0.02 |
| RAID-5 | 25.86 | (13.24-52.91) | 0.08 | (0.00-0.21) | 0.01 | 0.00 | 0.01 |
| RAID-6 | 36.05 | (5.55-87.84) | 0.16 | (0.00-0.85) | 0.01 | 0.00 | 0.02 |
| RAID-7 | 33.02 | (9.8-79.34) | 0.15 | (0.00-0.46) | 0.01 | 0.00 | 0.01 |

LM = Mean of Lagrange multiplier statistics across countries; Min/Max LM = lowest and highest LM statistics observed; p = mean of LM test p values ; range of p’s lowest and highest LM test p values observed; ES = Mean of item fit effect size statistics; Range of ES = lowest and highest Effect size statistics observed

Supplemental table 1 BRAF item parameters

| # | Item | Parameter | France | Germany | the Netherlands | Spain | Sweden | UK |
| --- | --- | --- | --- | --- | --- | --- | --- | --- |
| 1 | nrs | α | 0.919 | 1.024 | 0.919 | 0.767 | 0.919 | 1.171 |
| 1 | nrs | β1 | -2.675 | -2.389 | -2.675 | -1.589 | -2.675 | -3.474 |
| 1 | nrs | β2 | -2.042 | -2.2 | -2.042 | -1.852 | -2.042 | -0.892 |
| 1 | nrs | β3 | -1.311 | -1.468 | -1.311 | -0.781 | -1.311 | -1.476 |
| 1 | nrs | β4 | -0.832 | 0.047 | -0.832 | -0.38 | -0.832 | 0.106 |
| 1 | nrs | β5 | -0.256 | 0.487 | -0.256 | 0.319 | -0.256 | -0.705 |
| 1 | nrs | β6 | 0.235 | 0.607 | 0.235 | 0.128 | 0.235 | 0.633 |
| 1 | nrs | β7 | 0.345 | 1.313 | 0.345 | 0.562 | 0.345 | 0.537 |
| 1 | nrs | β8 | 1.774 | 1.39 | 1.774 | 0.989 | 1.774 | 1.555 |
| 1 | nrs | β9 | 2.787 | 2.337 | 2.787 | 1.746 | 2.787 | 2.869 |
| 1 | nrs | β10 | 3.269 | 3.153 | 3.269 | 3.341 | 3.269 | 3.174 |
| 2 | day | α | 0.47 | 0.546 | 0.47 | 0.519 | 0.64 | 0.741 |
| 2 | day | β1 | -0.814 | -0.934 | -0.814 | -0.304 | -1.622 | -2.183 |
| 2 | day | β2 | -1.274 | -1.418 | -1.274 | -1.25 | -1.125 | -0.346 |
| 2 | day | β3 | -0.687 | -0.596 | -0.687 | -0.65 | -0.321 | -0.809 |
| 2 | day | β4 | 0.188 | -0.213 | 0.188 | 0.534 | -0.353 | 0.045 |
| 2 | day | β5 | -0.049 | 0.477 | -0.049 | -0.195 | -0.29 | 0.08 |
| 2 | day | β6 | 1.269 | 1.803 | 1.269 | 1.248 | 1.297 | 2.042 |
| 2 | day | β7 | -2.558 | -2.993 | -2.558 | -1.494 | -2.725 | -1.897 |
| 3 | episode | α | 1.164 | 1.164 | 1.164 | 1.164 | 1.164 | 1.164 |
| 3 | episode | β1 | -1.283 | -1.283 | -1.283 | -1.283 | -1.283 | -1.283 |
| 3 | episode | β2 | 1.784 | 1.784 | 1.784 | 1.784 | 1.784 | 1.784 |
| 4 | physical | α | 1.993 | 1.993 | 1.993 | 1.993 | 1.993 | 1.993 |
| 4 | physical | β1 | -3.545 | -3.545 | -3.545 | -3.545 | -3.545 | -3.545 |
| 4 | physical | β2 | 0.763 | 0.763 | 0.763 | 0.763 | 0.763 | 0.763 |
| 4 | physical | β3 | 3.422 | 3.422 | 3.422 | 3.422 | 3.422 | 3.422 |
| 5 | bath | α | 1.436 | 1.436 | 1.699 | 1.436 | 1.436 | 1.436 |
| 5 | bath | β1 | 0.604 | 0.604 | -0.178 | 0.604 | 0.604 | 0.604 |
| 5 | bath | β2 | 2.492 | 2.492 | 1.948 | 2.492 | 2.492 | 2.492 |
| 5 | bath | β3 | 3.731 | 3.731 | 3.94 | 3.731 | 3.731 | 3.731 |
| 6 | dress | α | 1.335 | 1.335 | 1.452 | 1.335 | 1.335 | 1.335 |
| 6 | dress | β1 | 0.908 | 0.908 | 0.194 | 0.908 | 0.908 | 0.908 |
| 6 | dress | β2 | 2.596 | 2.596 | 2.006 | 2.596 | 2.596 | 2.596 |
| 6 | dress | β3 | 3.659 | 3.659 | 4.067 | 3.659 | 3.659 | 3.659 |
| 7 | work | α | 2.139 | 2.139 | 1.985 | 2.139 | 2.139 | 2.139 |
| 7 | work | β1 | -1.899 | -1.899 | -3.003 | -1.899 | -1.899 | -1.899 |
| 7 | work | β2 | 1.629 | 1.629 | 0.831 | 1.629 | 1.629 | 1.629 |
| 7 | work | β3 | 3.965 | 3.965 | 3.399 | 3.965 | 3.965 | 3.965 |
| 8 | avoid | α | 1.925 | 1.925 | 1.925 | 1.925 | 1.925 | 1.925 |
| 8 | avoid | β1 | -1.361 | -1.361 | -1.361 | -1.361 | -1.361 | -1.361 |
| 8 | avoid | β2 | 1.281 | 1.281 | 1.281 | 1.281 | 1.281 | 1.281 |
| 8 | avoid | β3 | 3.126 | 3.126 | 3.126 | 3.126 | 3.126 | 3.126 |
| 9 | social | α | 1.986 | 2.723 | 1.986 | 1.986 | 1.986 | 1.986 |
| 9 | social | β1 | -1.163 | 0.698 | -1.163 | -1.163 | -1.163 | -1.163 |
| 9 | social | β2 | 1.835 | 2.503 | 1.835 | 1.835 | 1.835 | 1.835 |
| 9 | social | β3 | 3.156 | 4.324 | 3.156 | 3.156 | 3.156 | 3.156 |
| 10 | cancel | α | 1.768 | 1.768 | 1.768 | 1.768 | 1.768 | 1.768 |
| 10 | cancel | β1 | -0.848 | -0.848 | -0.848 | -0.848 | -0.848 | -0.848 |
| 10 | cancel | β2 | 1.547 | 1.547 | 1.547 | 1.547 | 1.547 | 1.547 |
| 10 | cancel | β3 | 3.11 | 3.11 | 3.11 | 3.11 | 3.11 | 3.11 |
| 11 | refuse | α | 1.651 | 1.651 | 1.929 | 1.651 | 1.651 | 1.651 |
| 11 | refuse | β1 | 0.071 | 0.071 | 0.469 | 0.071 | 0.071 | 0.071 |
| 11 | refuse | β2 | 1.928 | 1.928 | 2.336 | 1.928 | 1.928 | 1.928 |
| 11 | refuse | β3 | 3.317 | 3.317 | 4.189 | 3.317 | 3.317 | 3.317 |
| 12 | mental | α | 1.888 | 1.888 | 1.888 | 1.888 | 1.945 | 1.888 |
| 12 | mental | β1 | -1.002 | -1.002 | -1.002 | -1.002 | -2.222 | -1.002 |
| 12 | mental | β2 | 1.952 | 1.952 | 1.952 | 1.952 | 1.611 | 1.952 |
| 12 | mental | β3 | 3.96 | 3.96 | 3.96 | 3.96 | 3.594 | 3.96 |
| 13 | forget | α | 1.611 | 1.611 | 1.611 | 1.611 | 1.611 | 1.611 |
| 13 | forget | β1 | -0.347 | -0.347 | -0.347 | -0.347 | -0.347 | -0.347 |
| 13 | forget | β2 | 2.116 | 2.116 | 2.116 | 2.116 | 2.116 | 2.116 |
| 13 | forget | β3 | 3.551 | 3.551 | 3.551 | 3.551 | 3.551 | 3.551 |
| 14 | think | α | 1.672 | 1.672 | 1.672 | 1.672 | 1.672 | 1.672 |
| 14 | think | β1 | -0.291 | -0.291 | -0.291 | -0.291 | -0.291 | -0.291 |
| 14 | think | β2 | 2.336 | 2.336 | 2.336 | 2.336 | 2.336 | 2.336 |
| 14 | think | β3 | 3.755 | 3.755 | 3.755 | 3.755 | 3.755 | 3.755 |
| 15 | concen | α | 1.83 | 1.83 | 1.83 | 1.83 | 1.83 | 1.83 |
| 15 | concen | β1 | -1.036 | -1.036 | -1.036 | -1.036 | -1.036 | -1.036 |
| 15 | concen | β2 | 2.172 | 2.172 | 2.172 | 2.172 | 2.172 | 2.172 |
| 15 | concen | β3 | 3.909 | 3.909 | 3.909 | 3.909 | 3.909 | 3.909 |
| 16 | mistake | α | 1.706 | 1.706 | 1.706 | 1.706 | 1.706 | 1.706 |
| 16 | mistake | β1 | 0.221 | 0.221 | 0.221 | 0.221 | 0.221 | 0.221 |
| 16 | mistake | β2 | 3.008 | 3.008 | 3.008 | 3.008 | 3.008 | 3.008 |
| 16 | mistake | β3 | 4.032 | 4.032 | 4.032 | 4.032 | 4.032 | 4.032 |
| 17 | control | α | 1.884 | 1.884 | 1.884 | 1.884 | 2.021 | 1.884 |
| 17 | control | β1 | -0.829 | -0.829 | -0.829 | -0.829 | -0.465 | -0.829 |
| 17 | control | β2 | 2.099 | 2.099 | 2.099 | 2.099 | 2.589 | 2.099 |
| 17 | control | β3 | 3.753 | 3.753 | 3.753 | 3.753 | 4.424 | 3.753 |
| 18 | emb | α | 1.557 | 1.552 | 1.552 | 1.273 | 1.392 | 1.552 |
| 18 | emb | β1 | -0.897 | 0.433 | 0.433 | 1.371 | 1.029 | 0.433 |
| 18 | emb | β2 | 1.814 | 2.53 | 2.53 | 2.537 | 2.644 | 2.53 |
| 18 | emb | β3 | 1.988 | 3.489 | 3.489 | 3.266 | 3.308 | 3.489 |
| 19 | upset | α | 1.471 | 1.471 | 1.471 | 1.471 | 2.036 | 1.471 |
| 19 | upset | β1 | -0.011 | -0.011 | -0.011 | -0.011 | -1.222 | -0.011 |
| 19 | upset | β2 | 1.828 | 1.828 | 1.828 | 1.828 | 2.039 | 1.828 |
| 19 | upset | β3 | 2.907 | 2.907 | 2.907 | 2.907 | 3.756 | 2.907 |
| 20 | depres | α | 1.432 | 1.432 | 1.432 | 1.432 | 1.432 | 1.432 |
| 20 | depres | β1 | -0.264 | -0.264 | -0.264 | -0.264 | -0.264 | -0.264 |
| 20 | depres | β2 | 1.743 | 1.743 | 1.743 | 1.743 | 1.743 | 1.743 |
| 20 | depres | β3 | 2.547 | 2.547 | 2.547 | 2.547 | 2.547 | 2.547 |

Supplemental table 2 RAID item parameters

| item | parameter | France | Germany | the Netherlands | Spain | Sweden | UK |
| --- | --- | --- | --- | --- | --- | --- | --- |
| RAID1 | α | 1.169 | 1.169 | 1.169 | 1.169 | 1.169 | 1.17 |
|  | β1 | -2.329 | -2.329 | -2.329 | -2.329 | -2.329 | -2.312 |
|  | β2 | -1.449 | -1.449 | -1.449 | -1.449 | -1.449 | -1.441 |
|  | β3 | -1.099 | -1.099 | -1.099 | -1.099 | -1.099 | -1.094 |
|  | β4 | 0.018 | 0.018 | 0.018 | 0.018 | 0.018 | 0.019 |
|  | β5 | 0.314 | 0.314 | 0.314 | 0.314 | 0.314 | 0.311 |
|  | β6 | 0.407 | 0.407 | 0.407 | 0.407 | 0.407 | 0.401 |
|  | β7 | 1.197 | 1.197 | 1.197 | 1.197 | 1.197 | 1.189 |
|  | β8 | 1.517 | 1.517 | 1.517 | 1.517 | 1.517 | 1.506 |
|  | β9 | 3.16 | 3.16 | 3.16 | 3.16 | 3.16 | 3.14 |
|  | β10 | 3.086 | 3.086 | 3.086 | 3.086 | 3.086 | 3.049 |
| RAID2 | α | 1.375 | 1.375 | 1.375 | 1.375 | 1.375 | 1.381 |
|  | β1 | -2.217 | -2.217 | -2.217 | -2.217 | -2.217 | -2.209 |
|  | β2 | -1.607 | -1.607 | -1.607 | -1.607 | -1.607 | -1.604 |
|  | β3 | -0.729 | -0.729 | -0.729 | -0.729 | -0.729 | -0.727 |
|  | β4 | -0.142 | -0.142 | -0.142 | -0.142 | -0.142 | -0.143 |
|  | β5 | 0.403 | 0.403 | 0.403 | 0.403 | 0.403 | 0.4 |
|  | β6 | 0.366 | 0.366 | 0.366 | 0.366 | 0.366 | 0.361 |
|  | β7 | 1.121 | 1.121 | 1.121 | 1.121 | 1.121 | 1.116 |
|  | β8 | 2.017 | 2.017 | 2.017 | 2.017 | 2.017 | 2.011 |
|  | β9 | 3.216 | 3.216 | 3.216 | 3.216 | 3.216 | 3.202 |
|  | β10 | 3.772 | 3.772 | 3.772 | 3.772 | 3.772 | 3.742 |
| RAID3 | α | 1.043 | 0.997 | 1.043 | 1.043 | 1.043 | 1.033 |
|  | β1 | -1.518 | -1.821 | -1.518 | -1.518 | -1.518 | -1.577 |
|  | β2 | -1.622 | -1.199 | -1.622 | -1.622 | -1.622 | -1.514 |
|  | β3 | -1.015 | -1.235 | -1.015 | -1.015 | -1.015 | -1.059 |
|  | β4 | -0.306 | 0.16 | -0.306 | -0.306 | -0.306 | -0.207 |
|  | β5 | 0.001 | 0.364 | 0.001 | 0.001 | 0.001 | 0.058 |
|  | β6 | 0.232 | 0.08 | 0.232 | 0.232 | 0.232 | 0.201 |
|  | β7 | 0.26 | 0.947 | 0.26 | 0.26 | 0.26 | 0.349 |
|  | β8 | 1.042 | 1.905 | 1.042 | 1.042 | 1.042 | 1.109 |
|  | β9 | 2.273 | 1.342 | 2.273 | 2.273 | 2.273 | 2.166 |
|  | β10 | 2.792 | 3.038 | 2.792 | 2.792 | 2.792 | 2.786 |
| RAID4 | α | 0.563 | 0.71 | 0.563 | 0.563 | 0.563 | 0.609 |
|  | β1 | -0.174 | -0.316 | -0.174 | -0.174 | -0.174 | -0.174 |
|  | β2 | -0.254 | -0.881 | -0.254 | -0.254 | -0.254 | -0.419 |
|  | β3 | -0.295 | -0.078 | -0.295 | -0.295 | -0.295 | -0.146 |
|  | β4 | 0.238 | 0.055 | 0.238 | 0.238 | 0.238 | 0.119 |
|  | β5 | 0.01 | -0.024 | 0.01 | 0.01 | 0.01 | 0.063 |
|  | β6 | 0.369 | 1.018 | 0.369 | 0.369 | 0.369 | 0.454 |
|  | β7 | 0.311 | -0.36 | 0.311 | 0.311 | 0.311 | 0.219 |
|  | β8 | 0.816 | 0.671 | 0.816 | 0.816 | 0.816 | 0.761 |
|  | β9 | 1.178 | 1.264 | 1.178 | 1.178 | 1.178 | 1.148 |
|  | β10 | 2.017 | 1.337 | 2.017 | 2.017 | 2.017 | 1.815 |
| RAID5 | α | 1.199 | 1.647 | 1.647 | 1.647 | 1.647 | 1.535 |
|  | β1 | -2.674 | -3.589 | -3.589 | -3.589 | -3.589 | -3.364 |
|  | β2 | -2.233 | -1.996 | -1.996 | -1.996 | -1.996 | -1.985 |
|  | β3 | -1.007 | -1.028 | -1.028 | -1.028 | -1.028 | -1.009 |
|  | β4 | 0.104 | -0.05 | -0.05 | -0.05 | -0.05 | -0.028 |
|  | β5 | -0.585 | 0.04 | 0.04 | 0.04 | 0.04 | -0.08 |
|  | β6 | 1.197 | 0.906 | 0.906 | 0.906 | 0.906 | 0.908 |
|  | β7 | 0.307 | 1.825 | 1.825 | 1.825 | 1.825 | 1.536 |
|  | β8 | 2.269 | 1.681 | 1.681 | 1.681 | 1.681 | 1.671 |
|  | β9 | 1.599 | 4.11 | 4.11 | 4.11 | 4.11 | 3.653 |
|  | β10 | 2.522 | 4.56 | 4.56 | 4.56 | 4.56 | 4.057 |
| RAID6 | α | 0.977 | 0.977 | 0.977 | 0.977 | 0.977 | 0.984 |
|  | β1 | -1.654 | -1.654 | -1.654 | -1.654 | -1.654 | -1.65 |
|  | β2 | -0.894 | -0.894 | -0.894 | -0.894 | -0.894 | -0.893 |
|  | β3 | -0.532 | -0.532 | -0.532 | -0.532 | -0.532 | -0.532 |
|  | β4 | 0.486 | 0.486 | 0.486 | 0.486 | 0.486 | 0.485 |
|  | β5 | -0.075 | -0.075 | -0.075 | -0.075 | -0.075 | -0.077 |
|  | β6 | 0.747 | 0.747 | 0.747 | 0.747 | 0.747 | 0.744 |
|  | β7 | 1.019 | 1.019 | 1.019 | 1.019 | 1.019 | 1.017 |
|  | β8 | 1.529 | 1.529 | 1.529 | 1.529 | 1.529 | 1.525 |
|  | β9 | 2.054 | 2.054 | 2.054 | 2.054 | 2.054 | 2.047 |
|  | β10 | 2.931 | 2.931 | 2.931 | 2.931 | 2.931 | 2.913 |
| RAID7 | α | 1.205 | 1.205 | 1.461 | 1.205 | 1.061 | 1.178 |
|  | β1 | -1.631 | -1.631 | -1.487 | -1.631 | -0.987 | -1.462 |
|  | β2 | -1.275 | -1.275 | -1.167 | -1.275 | -0.691 | -0.992 |
|  | β3 | -0.566 | -0.566 | -0.178 | -0.566 | -0.234 | -0.324 |
|  | β4 | 0.484 | 0.484 | 0.589 | 0.484 | 0.573 | 0.481 |
|  | β5 | 0.173 | 0.173 | 0.565 | 0.173 | 0.318 | 0.295 |
|  | β6 | 1.055 | 1.055 | 1.269 | 1.055 | 1.312 | 1.098 |
|  | β7 | 1.18 | 1.18 | 1.215 | 1.18 | 0.946 | 1.247 |
|  | β8 | 2.367 | 2.367 | 3.239 | 2.367 | 2.822 | 2.375 |
|  | β9 | 2.456 | 2.456 | 3.776 | 2.456 | 3.893 | 2.895 |
|  | β10 | 2.915 | 2.915 | 2.915 | 2.915 | 2.915 | 3.121 |
